# Start value of cerebral saturation in prehospital cardiac arrest patients: does it mean something?

**DOI:** 10.1186/cc12244

**Published:** 2013-03-19

**Authors:** C Genbrugge, I Meex, S Scheyltjens, J Dens, C De Deyne

**Affiliations:** 1 ZOL, Genk, Belgium

## Introduction

During cardiopulmonary resuscitation (CPR) monitoring possibilities are limited. Parnia and colleagues investigated the feasibility and role of near-infrared spectroscopy (NIRS) during CPR in cardiac arrest patients (CA) [1]. NIRS could have a role in predicting return of spontaneous circulation (ROSC). Recently, the Equanox^® ^with four wavelengths sensor was validated to provide absolute data on regional cerebral saturation [2]. We measured cerebral oxygenation (rSO_2_) during CPR with NIRS technology and analyzed the differences between initial cerebral saturations in patients achieving ROSC compared with patients without ROSC.

## Methods

With IRB approval, was measured with NIRS during rSO_2 _resuscitation in 18 out-of hospital CA patients. The Equanox^® ^Advance values, (NONIN), a NIRS monitoring device that measures absolute rSO_2 _was applied on the right side of the patient's forehead when the medical emergency team arrived in a resuscitation setting. Placement of the probe did not interfere with the advanced life support algorithm. The sensor remained on the patient's forehead during resuscitation, and if ROSC was reached the probe was removed on arrival at the emergency department. If ROSC was not achieved, the probe was removed prehospital. ROSC was defined as ROSC during more than 20 minutes. The Mann-Whitney test was utilized for comparison of survivor and nonsurvivor data. Student's *t *test was performed to compare the initial rSO_2_.

## Results

Of the 18 patients, nine patients had ROSC (survivors). The initial rhythm was the same in both groups, six patients in each group had asystole as initial rhythm. In the group of survivors were six female patients, in the nonsurvivors were two female patients. The mean age in ROSC and no-ROSC groups is respectively 75.8 years (SD ±12.8) and 69.4 years (SD ±22.9, *P*= 0.48). The mean rSO_2 _at arrival of the emergency medical team was 31.56% (SD ±29.4) and 12.78% (SD ±12.7) respectively in the ROSC group and no-ROSC group (*P *= 0.1). The mean time between collapse and start of CPR (basic life support of bystanders) was 6.9 minutes (SD ±8.2) in the no-ROSC group and 8.2 minutes (SD ±7.08, *P *= 0.69) in the ROSC group.

## Conclusion

Initial rSO_2 _values in out-of hospital CA patients with ROSC showed a tendency towards higher values compared with nonsurvivors, but no significant difference could be demonstrated, probably related to the small number of patients included in this preliminary report.
